# Genomic Variability of *Mycobacterium tuberculosis* Strains of the Euro-American Lineage Based on Large Sequence Deletions and 15-Locus MIRU-VNTR Polymorphism

**DOI:** 10.1371/journal.pone.0107150

**Published:** 2014-09-08

**Authors:** Laura Rindi, Chiara Medici, Nicola Bimbi, Andrea Buzzigoli, Nicoletta Lari, Carlo Garzelli

**Affiliations:** Dipartimento di Ricerca Traslazionale e delle Nuove Tecnologie in Medicina e Chirurgia, Università di Pisa, Pisa, Italy; Institut Pasteur de Lille, France

## Abstract

A sample of 260 *Mycobacterium tuberculosis* strains assigned to the Euro-American family was studied to identify phylogenetically informative genomic regions of difference (RD). Mutually exclusive deletions of regions RD115, RD122, RD174, RD182, RD183, RD193, RD219, RD726 and RD761 were found in 202 strains; the RD^Rio^ deletion was detected exclusively among the RD174-deleted strains. Although certain deletions were found more frequently in certain spoligotype families (i.e., deletion RD115 in T and LAM, RD174 in LAM, RD182 in Haarlem, RD219 in T and RD726 in the “Cameroon” family), the RD-defined sublineages did not specifically match with spoligotype-defined families, thus arguing against the use of spoligotyping for establishing exact phylogenetic relationships between strains. Notably, when tested for *katG*463/*gyrA*95 polymorphism, all the RD-defined sublineages belonged to Principal Genotypic Group (PGG) 2, except sublineage RD219 exclusively belonging to PGG3; the 58 Euro-American strains with no deletion were of either PGG2 or 3. A representative sample of 197 isolates was then analyzed by standard 15-locus MIRU-VNTR typing, a suitable approach to independently assess genetic relationships among the strains. Analysis of the MIRU-VNTR typing results by using a minimum spanning tree (MST) and a classical dendrogram showed groupings that were largely concordant with those obtained by RD-based analysis. Isolates of a given RD profile show, in addition to closely related MIRU-VNTR profiles, related spoligotype profiles that can serve as a basis for better spoligotype-based classification.

## Introduction

Comparative genomic studies have shown that the *Mycobacterium tuberculosis* complex has evolved through irreversible genetic events that occurred in ancient common progenitor strains [1]–[2]. Due to the virtual lack of horizontal genetic exchange between *M. tuberculosis* strains [2]–[3], mutations, deletions, and transpositions of chromosomal regions are considered the major forces driving *M. tuberculosis* genome evolution.

In the last decade, by whole-genome comparison of global samples of *M. tuberculosis* strains, a number of phylogenetically informative deletions of large genomic sequences have been identified [4], [5]. Large sequence polymorphisms (LSPs), together with additional, previously reported, phylogenetic markers, such as the regions of difference (RD) TbD1 and RD9 [6], the 7-bp deletion in the *pks* 15/1 locus [7], and the *katG*463/*gyrA*95 single nucleotide polymorphism (SNP) [5], allowed the recognition of six main phylogeographical lineages, i.e., Indo-Oceanic, East Asian, East African-Indian, Euro-American, West African 1 and West African 2 lineage, resulting from the evolution of the *M. tuberculosis* complex members in association to their host populations [5], [9]–[11]. In parallel, the discovery of the Direct Repeat (DR) locus polymorphism, based on the presence/absence of short spacer sequences connecting the DR sequences, and the development of the spacer oligonucleotide typing (spoligotyping) methodology [12], introduced a further tool for genetic analysis of the *M. tuberculosis* complex that soon became the most practiced typing system used in studies of genetic diversity [13]. In general, the polymorphisms probed by RD deletion analysis and spoligotyping turn out to be largely congruent, as they reflect the clonal population structure of the *M. tuberculosis* complex [5], [14]–[16], so that the spoligotype families are generally regarded as sublineages within the LSP-defined main lineages [17]; in certain cases, however, spoligotype homoplasy, *i.e.* identical molecular pattern of the DR locus in strains belonging to different evolutionary lineages, likely resulting from convergent evolution, has been reported [17]–[23].

The Euro-American lineage, that is the prevalent lineage in Europe and in the Americas and is also widespread across different regions of Africa and the Middle East, includes strains belonging to Principal Genotypic Groups (PGG) 2 and 3, according to the *katG*463*/gyrA*95 polymorphism classification [8]. The Euro-American lineage consists of 10 distinct sublineages, each defined by a large sequence deletion, named RD115, RD122, RD174 (that recently has been demonstrated to co-segregate with the so-called deletion RD^Rio^ [24]–[25]), RD182, RD183, RD193, RD219, RD724, RD726 and RD761, and an 11^th^ group including strains with no RD deletion (the so-called H37Rv-like) [5]. By spoligotyping, the Euro-American lineage, is characterized by deletion of spacers 33–36 in the DR locus [17], [26] and consists of the major families T, Haarlem (H), Latin American-Mediterranean (LAM), S and X, including a total of 34 subfamilies.

In this paper we report the genotypic characterization of a collection of Euro-American strains recently isolated in Tuscany, Italy, a region with a low prevalence of tuberculosis (TB), but where, due to the immigration from high-prevalence TB countries [27], the ethnic diversity of TB patients provides an opportunity to study a global sample of Euro-American strains. In particular, the purposes of this paper are: (i) to identify the deletions of specific large genomic sequences in *M. tuberculosis* isolates assigned to the spoligotype families included in the Euro-American lineage; (ii) to determine the polymorphism of DNA minisatellites of these isolates by the 15-locus Mycobacterial Interspersed Repetitive Units - Variable Number of Tandem Repeats (MIRU-VNTR) assay, a high-throughput typing tool that is becoming the international gold standard for typing of *M. tuberculosis* isolates offering a discriminatory power that practically equals that of IS*6110* RFLP typing system [28]; (iii) to gain new insights into the previously proposed phylogeny of the Euro-American lineage.

## Materials and Methods

### *M. tuberculosis* study strains

A set of 260 *M. tuberculosis* strains were selected from a collection of 780 strains of the Euro-American lineage isolated in Tuscany, Italy, during a 4-year period, from the same number of TB patients living in Tuscany, Italy, and admitted to 10 major community hospitals in the region. Study strains were selected taking care, whenever possible, to respect the proportion between Italian-born and foreign-born patients and to include strains from as many different patient's countries of birth as possible. One hundred fifty five isolates were from Italian-born patients, 105 from patients born in a total of 26 different countries.

### Spoligotyping

Spoligotyping analysis of isolates was performed basically as described by Kamerbeek et al. [12] using genomic DNA extracted from the bacteria grown on ADC-supplemented Middlebrook 7H9 or Lowenstein-Jensen medium by the cetyltrimethylammonium bromide (CTAB) method. The study strains were assigned to the spoligotype families on the basis of the spoligotype profiles defined in the last released publicly available spoligotype SITVITWEB database [29] and included 73 T isolates, 29 Haarlem isolates, 121 LAM isolates (which also comprise 10 isolates defined in this paper "LAM-like", as their spoligotype profiles are not present in the SITVITWEB database, although they show the typical spacer deletions of the LAM family), 16 Cameroon isolates (referred to in the SITVITWEB database as LAM10_CAM), 8 S isolates, and 13× isolates.

### Determination of RD deletions

A PCR-based method using specific primers was used to determine the presence or absence of the regions RD115, RD122, RD174, RD182, RD183, RD193, RD219, RD724, RD726 and RD761 [5]. All PCR reactions were performed in 0.5 ml-microcentrifuge reaction tubes in a final volume of 50 µl containing 10 mM Tris-HCl (pH 8.3), 2.0 mM MgCl_2_, 50 mM KCl, 0.1% Triton X-100, 5% DMSO, 0.5 µM each primer, 0.2 mM deoxynucleoside triphosphates, 1.25 U Taq polymerase (Dynazyme) and 2.0 µl bacterial DNA extracted as described above. PCR amplification was performed for one 2-min cycle at 95°C and 35 cycles of 30 sec at 94°C, 1 min at 64°C and 3 min at 72°C. The PCR products were visualised on 1–2% agarose gels stained with ethidium bromide.

Isolates bearing the RD174 deletion were also tested for the RD^Rio^ deletion by a multiplex PCR assay, as described by Gibson et al. [30].

### Mycobacterial Interspersed Repetitive Units - Variable Number of Tandem Repeats (MIRU-VNTR) typing

MIRU-VNTR typing was performed by PCR amplification of the following 15 loci, as described by Supply et al. [28]: 424, 577, 580, 802, 960, 1644, 1955, 2163, 2165, 2401, 2996, 3192, 3690, 4052, and 4156. The PCR fragments were analyzed by gel electrophoresis using 2% NuSieve agarose (Cambrex Bio Science Rockland). For each locus, sizes of amplicons were estimated by comparison with 20 bp and 100 bp markers (Superladder-low; GenSura, CA, USA) and the numbers of repetitive units were calculated on the basis of conventions previously reported [31]. MIRU-VNTR profile is expressed as a string of 15 numbers, each representing the number of tandem repeats (TR) at a given VNTR position, in the order given above. MIRU-VNTR data were analyzed by the MIRU-VNTR*plus* web application available at www.miru-vntrplus.org [32]–[33].

### Allelic diversity and genetic distance analysis

The allelic diversity (*h*) of the VNTR loci was calculated using the equation *h* = 1 – Σ *x_i_* ^2^ {*n*/(*n* – 1)} where *n* is the number of isolates and *x_i_* the frequency of the *i* ^th^ allele at the locus [34].

The genetic relationships among the MIRU-VNTR-typed isolates were analyzed by constructing a minimum spanning tree (MST), an undirected network in which all the MIRU-VNTR profiles within the population studied are linked together with the smallest possible linkages between nearest neighbours, by the MIRU-VNTR*plus* web application available at www.miru-vntrplus.org.

A dendrogram of genetic relationships was generated using the unweighted pair group method with arithmetic averages (UPGMA), by the MIRU-VNTR*plus* web application available at www.miru-vntrplus.org.

### Principal Genotypic Groups

The Principal Genotypic Groups (PGG) of study strains, defined on the basis of *katG*463 and *gyrA*95 allele polymorphism [8], were determined by a real-time PCR assay by the LightCycler instrument (Roche Applied Science, Germany), as previously reported [35].

## Results and Discussion

### 1. RD deletions among Euro-American *M. tuberculosis* isolates

A collection of 260 *M. tuberculosis* isolates of the Euro-American lineage assigned to the spoligotype-defined families T, Haarlem, LAM, Cameroon, S and X was studied by PCR to determine the presence or absence of regions RD115, RD122, RD174, RD182, RD183, RD193, RD219, RD724, RD726 and RD761. LAM isolates bearing the RD174 deletion were also tested for the RD^Rio^ deletion that it is known to co-segregate with the RD174 deletion [24]–[25].

Mutually exclusive RD deletions were found in each of 202 strains; no deletion was found in the remaining 58 strains (the so-called H37Rv-like strains). Deletion of RD724, typical of strains from Central Africa [5], was not found in the study set. Table 1 reports the distribution of the RD deletions among the spoligotype-defined families. In particular, deletion RD115 was found almost exclusively in isolates assigned to the spoligotype families T (15/53, 28.3%) and LAM (37/53, 69.8%). Deletion RD174 was found in 40 isolates, 39 of which (97.5%) belonging to the LAM family; these RD174-deleted LAM isolates and, as controls, 25 randomly selected LAM isolates without the RD174 deletion were also tested for the RD^Rio^ deletion. As expected, the RD^Rio^ deletion was detected exclusively among the RD174-deleted strains; in particular, as shown in Table 1, 37 out of 39 (94.9%) RD174-deleted isolates also showed the RD^Rio^ deletion. These results are partially in agreement with the data recently reported by Mokrousov and colleagues [36], showing the presence of RD^Rio^ deletion in all the RD174-deleted strains and the mutually exclusive occurrence of RD174/RD^Rio^ and RD115 deletion among the LAM family strains. Deletion RD182 was highly prevalent in the Haarlem family (27/32, 84.4%), but was also found in isolates assigned to LAM (3/32, 9.4%) and S (2/32, 6.3%) families. Deletion RD219 was found in a consistent proportion of isolates assigned to the T family; isolates bearing deletion RD219 were 91.3% (42/46) T and 8.7% (4/46) LAM. Noteworthy, deletion RD726 was highly prevalent (15/19, 78.9%) in isolates of the "Cameroon" family [37]–[38], reported in the SITVITWEB database as LAM10_CAM; the deletion was also found in 4 LAM isolates with spoligotype profiles similar to those typical of the LAM10_CAM (Cameroon) family, as they showed either one-spacer difference or deletion of spacers 21–24, instead of 23–25, typical of "Cameroon" strains [38] (data not shown). Notably, in three cases of these “Cameroon-like” strains, the isolates were from patients born in West Africa (Senegal, Nigeria, Ivory Coast), i.e., the geographic area of the typical Cameroon strains; the fourth one was an Italian-born patient. Deletion of RD726 can be reasonably considered a specific marker of the Cameroon family. Finally, certain RD deletions were found in a small number of isolates; in particular, deletions RD183 and RD193 were detected exclusively in isolates assigned to the X spoligotype family (3 and 7 isolates, respectively); similarly, deletion RD761 was found in a single isolate, assigned to the LAM family.

**Table 1:** Distribution of region of (genomic) differences (RD) deletions in 260 isolates of spoligotype families of the Euro-American lineage.

Spoligotype family^a^	No. of isolates with RD deletion										
	RD115	RD122	RD174 (RD^Rio^)^b^	RD182	RD183	RD193	RD219	RD724	RD726	RD761	None^c^
T (73)	15	1	-	-	-	-	42	-	-	-	15
Haarlem (29)	-	-	-	27	-	-	-	-	-	-	2
Latin American- Mediterranean (121)	37	-	39 (37)	3	-	-	4	-	4	1	33
Cameroon (16) (LAM10_CAM)	1	-	-	-	-	-	-	-	15	-	-
S (8)	-	-	1 (0)	2	-	-	-	-	-	-	5
X (13)	-	-	-	-	3	7	-	-	-	-	3
Total	53	1	40	32	3	7	46	-	19	1	58
(%)	(20.4)	(0.4)	(15.4)	(12.3)	(1.2)	(2.7)	(17.7)	(0.0)	(7.3)	(0.4)	(22.3)

a Isolates were assigned to spoligotype families on the basis of SITVITWEB database [29]. Total numbers of isolates in each family are bracketed.

b Numbers of isolates bearing the RD^Rio^ deletion are bracketed.

c" H37Rv-like" strains.

In all, although certain deletions were found more frequently in certain spoligotype families (i.e., deletion RD115 in T and LAM, RD174 in LAM, RD182 in Haarlem, RD219 in T family), the classification based on large sequence deletions, that are unique event polymorphisms and robust phylogenetic markers, shows a general lack of concordance with the classification proposed by spoligotyping. This reinforces the existing doubts on the ability of spoligotyping alone to reveal exact phylogenetic relationships between *M. tuberculosis* strains, particularly those of the evolutionary recent TbD1^–^/PGG2/3 Euro-American lineage [17], [39]–[41].

### 2. Principal Genotypic Groups (PGG) of RD-defined sublineages

When tested for *katG*463/*gyrA*95 polymorphism, the strains of all the RD-defined sublineages, with the exception of the strains of the sublineage RD219, belonged to PGG2. The 46 strains with the RD219 deletion belonged to PGG3. The remaining 58 H37Rv-like strains were either of PGG2 or PGG3.

### 3. MIRU-VNTR analysis of Euro-American *M. tuberculosis* isolates

We then determined the genetic diversity of our sample of Euro-American isolates by analysing the polymorphism of MIRU-VNTR loci, using the conventional 15-locus assay, in order to see whether this analysis fits better with the overall evolutionary picture provided by RD deletion and *katG*463/*gyrA*95 polymorphisms. In *M. tuberculosis*, in fact, the evolution of the MIRU-VNTR loci is estimated to occur at an average mutation rate of 10^−4^ per year per locus [15], a property that makes MIRU-VNTR loci suitable for phylogenetic analysis and classification, especially when complemented by other robust phylogenetic markers. For this purpose, a set of 197 of the 260 clinical isolates, including 28 isolates with deletion RD115, 40 with deletion RD174, 26 with deletion RD182, 3 with deletion RD183, 7 with deletion RD193, 10 with deletion RD726, 1 with deletion RD122, 42 with deletion RD219 and 40 with no deletion, were analyzed for the 15 locus-based MIRU-VNTR polymorphism as described by Supply et al. [28].

#### 3.1 Allelic diversity of isolates

We first considered the allelic variability of each MIRU-VNTR locus of the whole strain collection, independently of the RD-defined sublineages. As summarized in Table 2, the number of alleles at each MIRU-VNTR locus ranged between 4 (for loci MIRU 16, ETR-A, VNTR 47, MIRU 31) and 9 (for locus QUB-26). The allelic diversity (*h*) was in general high (0.38≤*h*≤0.83), with the exception of locus MIRU 04 that showed 2 repeat units in 94.4% of isolates, yielding *h* = 0.10, which indicates that locus MIRU 04 is highly conserved in the Euro-American isolate collection. However, when the RD-defined lineages were considered individually it became evident that certain MIRU-VNTR loci were highly conserved in certain lineages and variable in others (data not shown).

**Table 2:** Determination of allelic diversity at each MIRU-VNTR locus of 197 isolates of the Euro-American lineage.

TR copies^a^	Number of isolates at MIRU-VNTR locus														
	VNTR 42	VNTR 43	MIRU 04	MIRU 40	MIRU 10	MIRU 16	1955	QUB-11b	ETR-A	VNTR 47	MIRU 26	MIRU 31	VNTR 52	QUB-26	VNTR 53
0	18		1										5		18
1	79	2	2	58		15	9	3	2	65	19		12	5	37
2	73	14	186	35	6	55	74	41	111	84	21	38	79	5	93
3	10	41	7	43	76	115	102	51	71	1	14	150	83	8	40
4	5	130		50	91	10	9	67	13	47	33	8	10	24	3
5		8	1	6	21		3	20			82	1	8	52	
6				4	2			13			8			37	
7					1			1			17			32	
8											2			18	
9				1										11	
U^b^	12	2				2		1			1			5	6
Allelic diversity	0.65	0.50	0.10	0.77	0.62	0.56	0.58	0.76	0.55	0.65	0.76	0.38	0.65	0.83	0.67

a Numbers of repeat units at the MIRU-VNTR locus.

b U, undetermined (no PCR product was obtained; not considered for calculation of allelic diversity.

#### 3.2 Genetic relationships among the isolates

**Figure 1:**
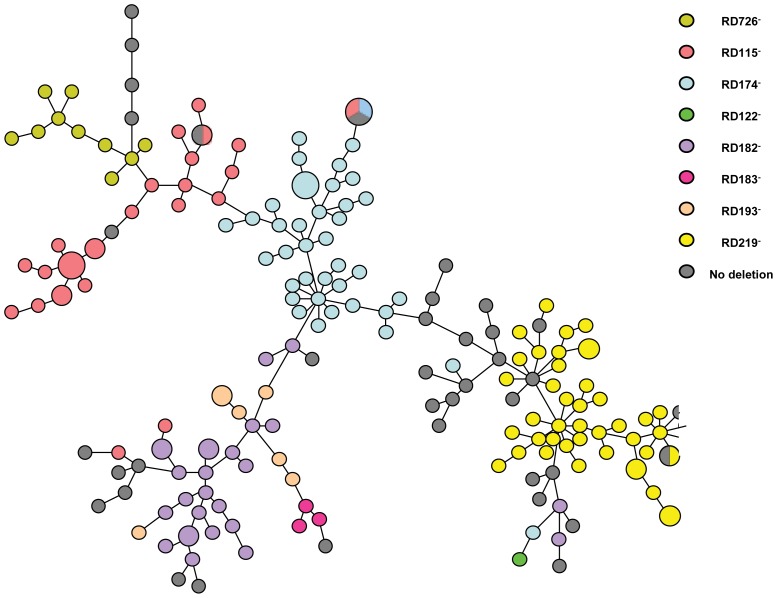
Minimum spanning tree based on MIRU-VNTR profiles of a set of 15 loci (424, 577, 580, 802, 960, 1644, 1955, 2163, 2165, 2401, 2996, 3192, 3690, 4052, and 4156) of *M. tuberculosis* clinical isolates bearing deletions RD115, RD122, RD 174, RD182, RD183, RD193, RD219 and RD726 (respectively indicated in different colours in the legend at the right side of the figure) and in isolates with no deletion (dark grey). Each small-size circle represents a single isolate; larger circles represent clusters of 2 or 3 isolates, depending on the circle size, with identical MIRU-VNTR profiles; larger circles with more than one colour represent clusters including 2 or 3 isolates with identical MIRU-VNTR profiles but differing in RD deletions. The length of the lines is not proportional to the number of allelic variation between the isolates; an on-line supplemented file (Figure S1) is provided to visualize the allelic differences on the connecting lines.

The genetic relationships among the 197 MIRU-VNTR-typed isolates were then visualized by constructing a MST, illustrated in Figure 1, constructed by the MIRU-VNTR*plus* web application available at www.miru-vntrplus.org. In our collection, 166 isolates showed unique MIRU-VNTR profiles and clusters of 2–3 isolates with identical MIRU-VNTR profiles were detected in a few cases; notably, three clusters of isolates yielding identical MIRU-VNTR profiles included isolates differing in RD deletions, which suggests the possibility of homoplasy also at the MIRU-VNTR level, as shown by others [42]–[43].

In general, our MST analysis shows that isolates bearing a given RD deletion tend to be grouped together, thus reflecting their minimal allelic variation when mutually compared and supporting a common recent ancestor; the isolates with no deletion (i.e., the H37Rv-like isolates) were scattered along the whole tree.

**Figure 2:**
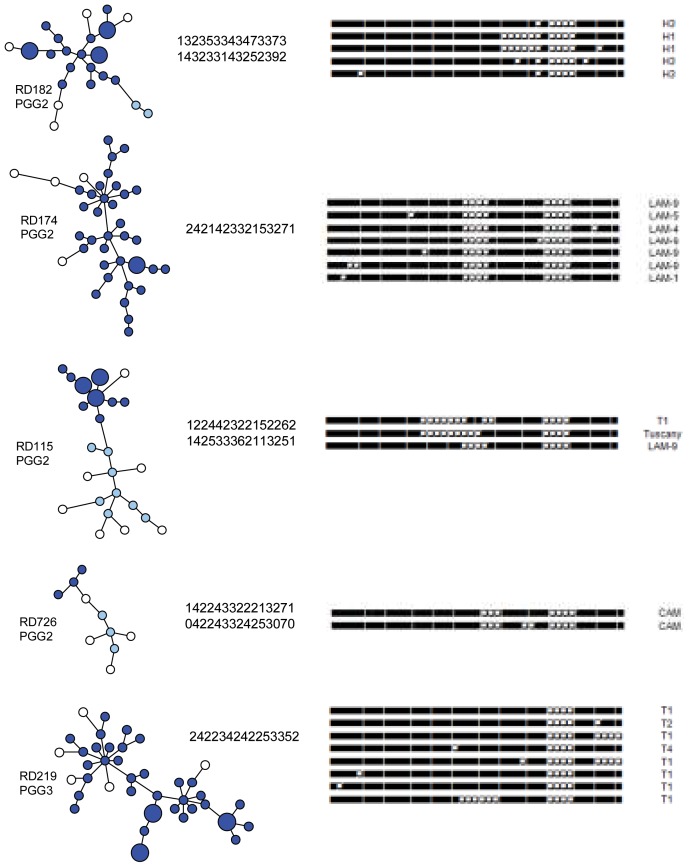
Description of the five major RD-defined sublineages of the Euro-American lineage based on *katG*463/*gyrA*95, 15-locus MIRU-VNTR and spoligotype polymorphism. For each RD-defined sublineage, a MST was constructed; the length of the lines is not proportional to the number of allelic variation between the isolates; an on-line supplemented file (Figure S2) is provided to visualize the allelic differences on the connecting lines. The RD deletion and the Principal Genotypic Group (PGG), defined by *katG*463/*gyrA*95 polymorphism, are reported at the left of each MST. Dark and light blue circles represent strains included in clonal complexes defined by a maximum of three locus differences; white circles represent strains outside the clonal complexes. Larger circles represent clusters of 2–3 isolates. At the right of each MST, the 15-locus MIRU-VNTR profile(s) of the central node strain(s) and the prevalent spoligotype patters (i.e, shared by at least 2 isolates) are provided; spoligotype patterns are shown in the conventional binary format; the spoligotype families, assigned to isolates on the basis of SITVITWEB database [29], are also shown at the right.

To directly visualize the MIRU-VNTR-based genetic relationships among the strains of each RD-defined Euro-American sublineage, a MST was constructed for each sublineage and the number of clonal complexes, arbitrarily defined as sets of strains yielding MIRU-VNTR profiles with a maximal of three locus differences, was determined. These results, integrated by data on the *katG*463/*gyrA*95 polymorphism and spoligotype profiles, are summarized in Figure 2. In particular, *Sublineage RD182*: The RD182-defined sublineage shows two distinct clonal complex, including 19 and 2 isolates, respectively, all belonging to PGG2. The first clonal complex consists of strains assigned to the Haarlem spoligotype subfamilies H1 and H3; the 2 strains of the second clonal complex were assigned to the S spoligotype family.

*Sublineage RD174*: The RD174-defined sublineage is characterized by a single large clonal complex including 36 strains. Such strains, all belonging to PGG2, were assigned to LAM spoligotype family (LAM1, LAM4, LAM5, LAM9 and LAM11_ZWE) with the exception of 2 isolates with undetermined spoligotype profiles (not shown). The 37 RD174-deleted isolates bearing the RD^Rio^ deletion were distributed among all the LAM subfamilies and also included 2 strains of undetermined spoligotype profile. The 3 RD174-deleted strains without the RD^Rio^ deletion were assigned to S, U and LAM5 spoligotype families, and only one of these (LAM5) was included in the clonal complex.

*Sublineage RD115*: The RD115-defined sublineage consists of two distinct clonal complexes, including 13 and 8 isolates, respectively, all belonging to PGG2. The first clonal complex includes strains of the spoligotype families T1 and T-Tuscany, and one LAM9 strain. The second clonal complex includes strains assigned to LAM spoligotype family (LAM1, LAM5, LAM9).

The distribution of LAM spoligotype families among different RD sublineages, particularly RD174 and RD115, supports the genetic inconsistencies of LAM spoligotype classifications, as also previously suggested [41].

*Sublineage RD726*: The RD726 lineage yielded 2 distinct clonal complex, each including 3 isolates, all belonging to PGG2, with spoligotype profiles typical of the Cameroon family, with the exception of one LAM9 isolate included in the first clonal complex; three of the strains outside of the complexes had spoligotype profiles typical of the Cameroon family and one showed an undetermined spoligotype profile.

*Sublineage RD219*: The RD219-defined sublineage is characterized by a single large clonal complex including 37 isolates, assigned to the T spoligotype family (T1, T2, T4, T-Tuscany), with the exception of one isolate with undetermined spoligotype profile. Notably, all RD219-deleted strains belong to PGG3.

*Sublineages RD193 and RD183*: The MIRU-VNTR analysis of the RD193-defined sublineage showed two distinct clonal complexes, including 3 and 2 isolates, respectively, all belonging to PGG2. The first clonal complex consists of strains assigned to the ×3 spoligotype subfamily; the second one includes strains assigned to the ×1 spoligotype subfamily. Two strains outside of the complexes were also assigned to ×1 spoligotype subfamily. The RD183-defined sublineage consists of 3 strains, all belonging to PGG2 and with spoligotype profiles ×2, grouped in a single clonal complex.

*H37Rv-like isolates*: The H37Rv-like isolates, i.e., isolates with no deletion, constitute a genotypically heterogeneous group that includes isolates of PGG2 or 3. If analyzed by MST according to the criteria reported above, six different MIRU-VNTR clonal complexes are detected; by spoligotyping, the H37Rv-like group includes isolates assigned to T, LAM, Haarlem, X and S families.

A dendrogram based on combined MIRU-VNTR and spoligotyping profiles was then constructed using the UPGMA method (Figure S3); for each isolate, data on RD deletions, SIT number and spoligotype subfamily are also included. This analysis emphasizes that isolates bearing a certain RD deletion tend to be clustered in the dendrogram, and show, in addition to closely related MIRU-VNTR profiles, related spoligotype profiles that define the families/subfamilies of the spoligotype-based classification. For example, the isolates bearing deletion RD219 constitute a discrete group of related strains, all belonging to spoligotype family T, and segregate at one side of the dendrogram together with H37Rv-like isolates of spoligotype family T. Similarly, isolates bearing deletions RD182, RD174 and RD726 cluster in discrete groups of strains, each one with closely related MIRU-VNTR profiles and spoligotypes typical, respectively, of the Haarlem, LAM, and Cameroon (LAM10_CAM) families. Conversely, isolates bearing the RD115 deletion cluster in two groups distant in the dendrogram, each one including isolates with related MIRU-VNTR profiles, but with spoligotype profiles typical of T and LAM isolates, respectively.

## Concluding Remarks

Our analysis of a global sample of Euro-American isolates, based on robust phylogenetic markers such as *katG*463/*gyrA*95 SNP and deletions of large RD genomic sequences, provides a framework of the genomic diversity of the *M. tuberculosis* strains that constitute the Euro-American lineage. The analysis of the polymorphism of the MIRU-VNTR loci is largely concordant with that obtained by RD-based analysis, as isolates of a given RD profile show closely related MIRU-VNTR profiles that might also serve as a basis for better spoligotype-based classification, especially when complemented by other robust phylogenetic markers. Moreover, as evolution of the MIRU-VNTR loci is estimated to occur at slow mutation rates [15], making them suitable for inferring long-term evolutionary histories, MIRU-VNTR analysis can provide a suitable tool for phylogenetic analysis of the Euro-American lineage.

## Supporting Information

Figure S1 **Printout of MST shown in** **Figure 1** **in which the numbers of allelic differences are shown on the connecting lines.** Each empty circle represents a single isolate; dark and light blue circles represent clusters of 3 or 2 isolates, respectively, with identical MIRU-VNTR profiles.
(PDF)

Figure S2 **Printouts of the MSTs shown in** **Figure 2** **in which the numbers of allelic differences are shown on the connecting lines.** Each empty circle represents a single isolate; dark and light gray circles represent clusters of 3 or 2 isolates, respectively, with identical MIRU-VNTR profiles.
(PDF)

Figure S3 **Dendrogram based on combined MIRU-VNTR and spoligotyping data from 197 Euro-American** ***M. tuberculosis*** **isolates bearing deletions RD115, RD122, RD 174, RD182, RD183, RD193, RD219 and RD726 or with no deletion.** The dendrogram was generated using the UPGMA method by the MIRU-VNTR*plus* web application available at www.miru-vntrplus.org. The columns 1 to 6 on the right of the dendrogram represent respectively: 1) isolate ID code (boxed); 2) RD deletion; 3) the SIT (Spoligotype International Type) number; 4) the spoligotype subfamily; 5) the VNTR profiles expressed as a string of 15 numbers, each representing the number of tandem repeats (TR) at a given VNTR position, in the order stated in the paper; 6) the spoligotype binary profile, given as black and white boxes indicating the presence and absence, respectively, of the specific spacer at position 1 to 43 in the DR locus.
(PDF)
