# Correction: Genomic Variability of *Mycobacterium tuberculosis* Strains of the Euro-American Lineage Based on Large Sequence Deletions and 15-Locus MIRU-VNTR Polymorphism

**DOI:** 10.1371/journal.pone.0114676

**Published:** 2014-11-24

**Authors:** The PLOS ONE Staff

Figure 1 is erroneously cropped. Please refer to a complete version here.

**Figure 1:**
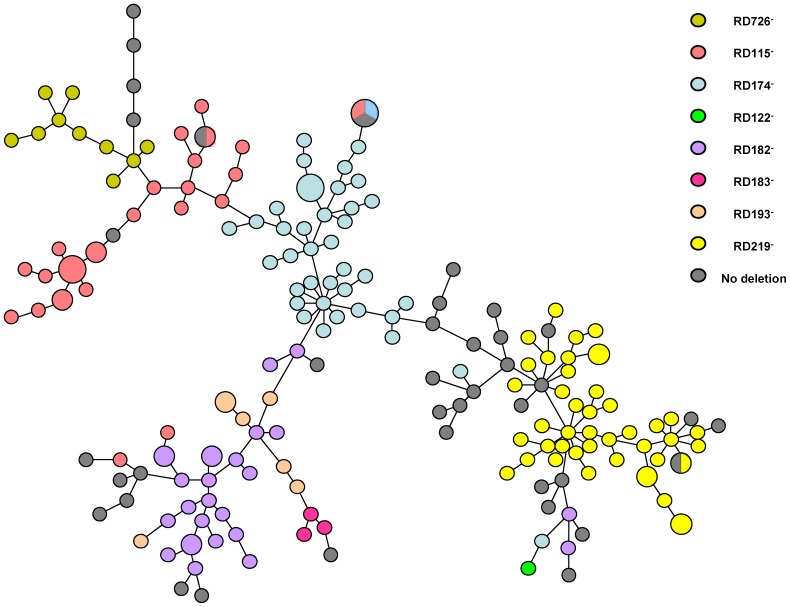
Minimum spanning tree based on MIRU-VNTR profiles of a set of 15 loci (424, 577, 580, 802, 960, 1644, 1955, 2163, 2165, 2401, 2996, 3192, 3690, 4052, and 4156) of M. tuberculosis clinical isolates bearing deletions RD115, RD122, RD 174, RD182, RD183, RD193, RD219 and RD726 (respectively indicated in different colours in the legend at the right side of the figure) and in isolates with no deletion (dark grey). Each small-size circle represents a single isolate; larger circles represent clusters of 2 or 3 isolates, depending on the circle size, with identical MIRU-VNTR profiles; larger circles with more than one colour represent clusters including 2 or 3 isolates with identical MIRU-VNTR profiles but differing in RD deletions. The length of the lines is not proportional to the number of allelic variation between the isolates; an on-line supplemented file (Figure S1) is provided to visualize the allelic differences on the connecting lines.
