# Supplementary figures and images for: Genomic Variability of Mycobacterium tuberculosis Strains of the Euro-American Lineage Based on Large Sequence Deletions and 15-Locus MIRU-VNTR Polymorphism
Source: PLoS One. 2014 Sep 8;9(9):e107150. doi: 10.1371/journal.pone.0107150 (PMC4157836; doi:10.1371/journal.pone.0107150)

Figure S1

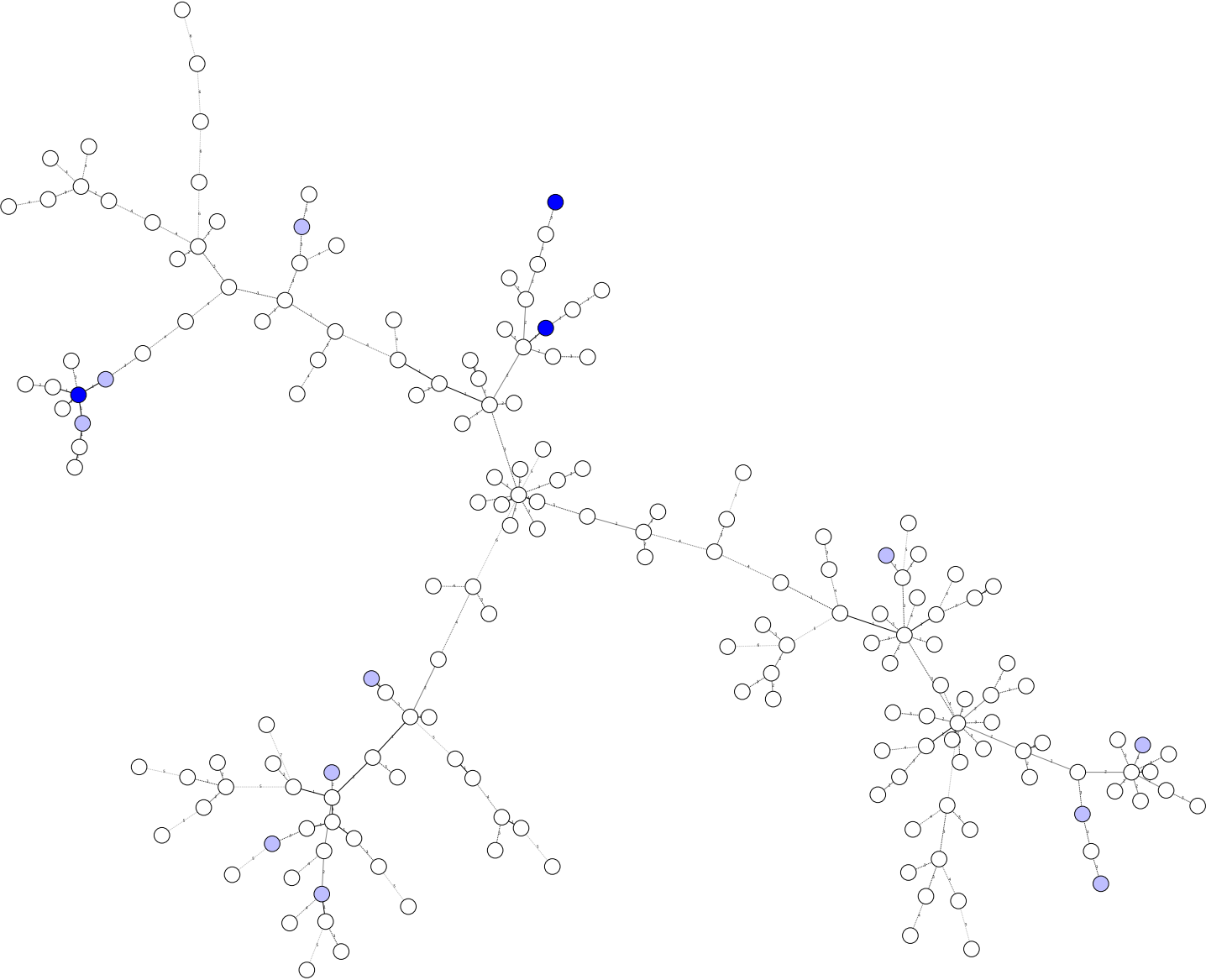

Supplement: Figure S1 — Printout of MST shown in Figure 1 in which the numbers of allelic differences are shown on the connecting lines. Each empty circle represents a single isolate; dark and light blue circles represent clusters of 3 or 2 isolates, respectively, with identical MIRU-VNTR profiles. (PDF) [file pone.0107150.s001.pdf]

Figure S2

**RD182**

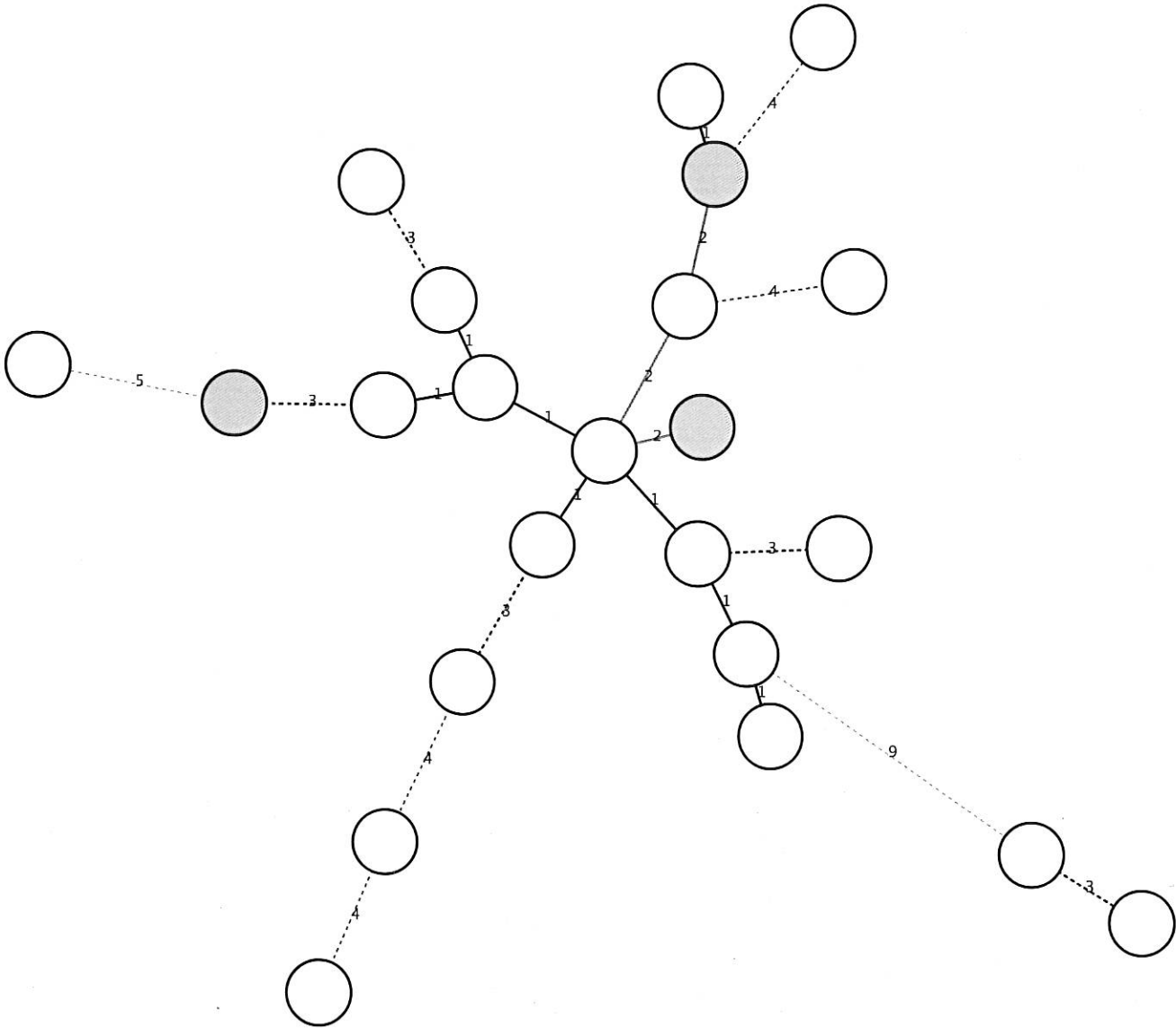

RD174

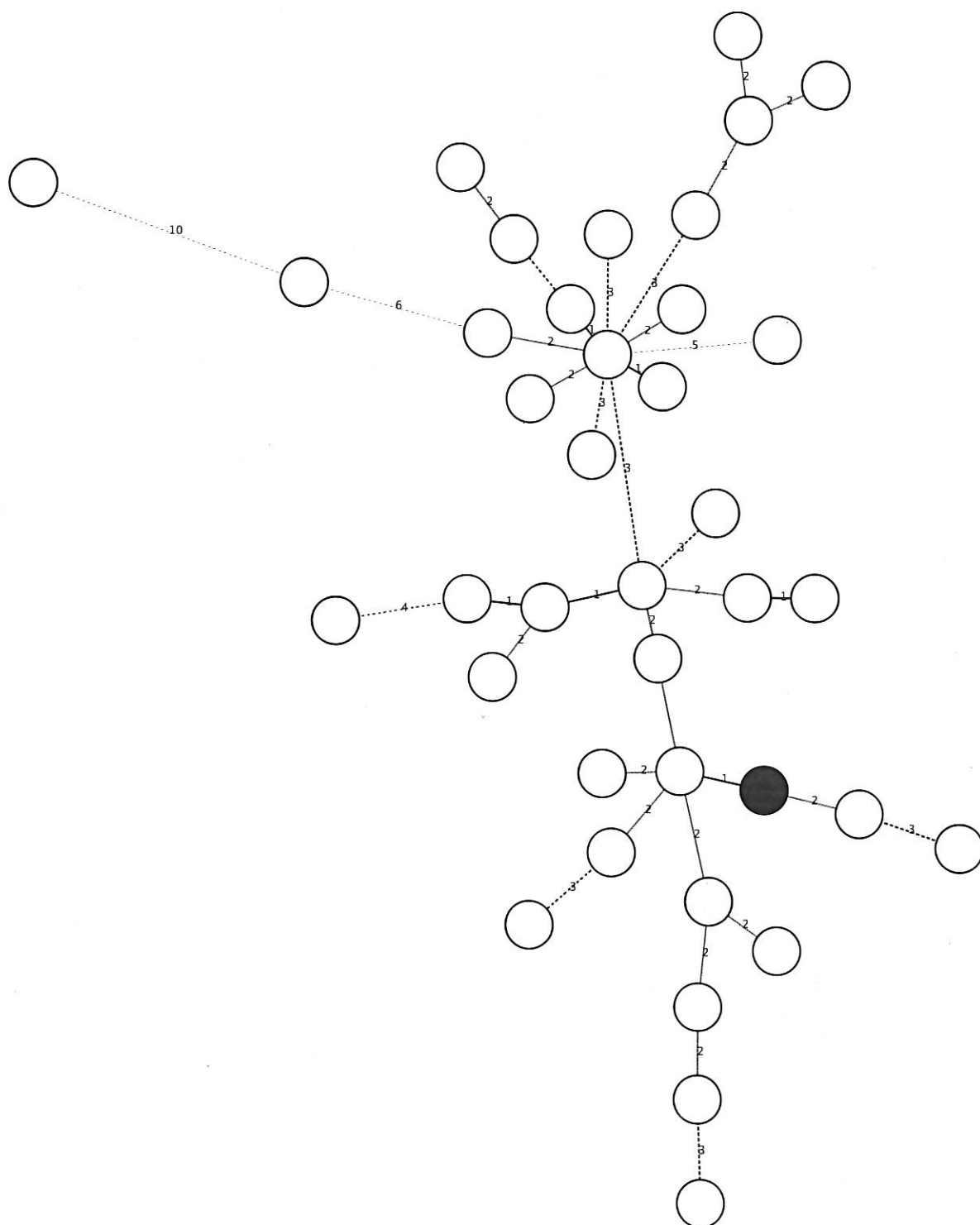

RD115

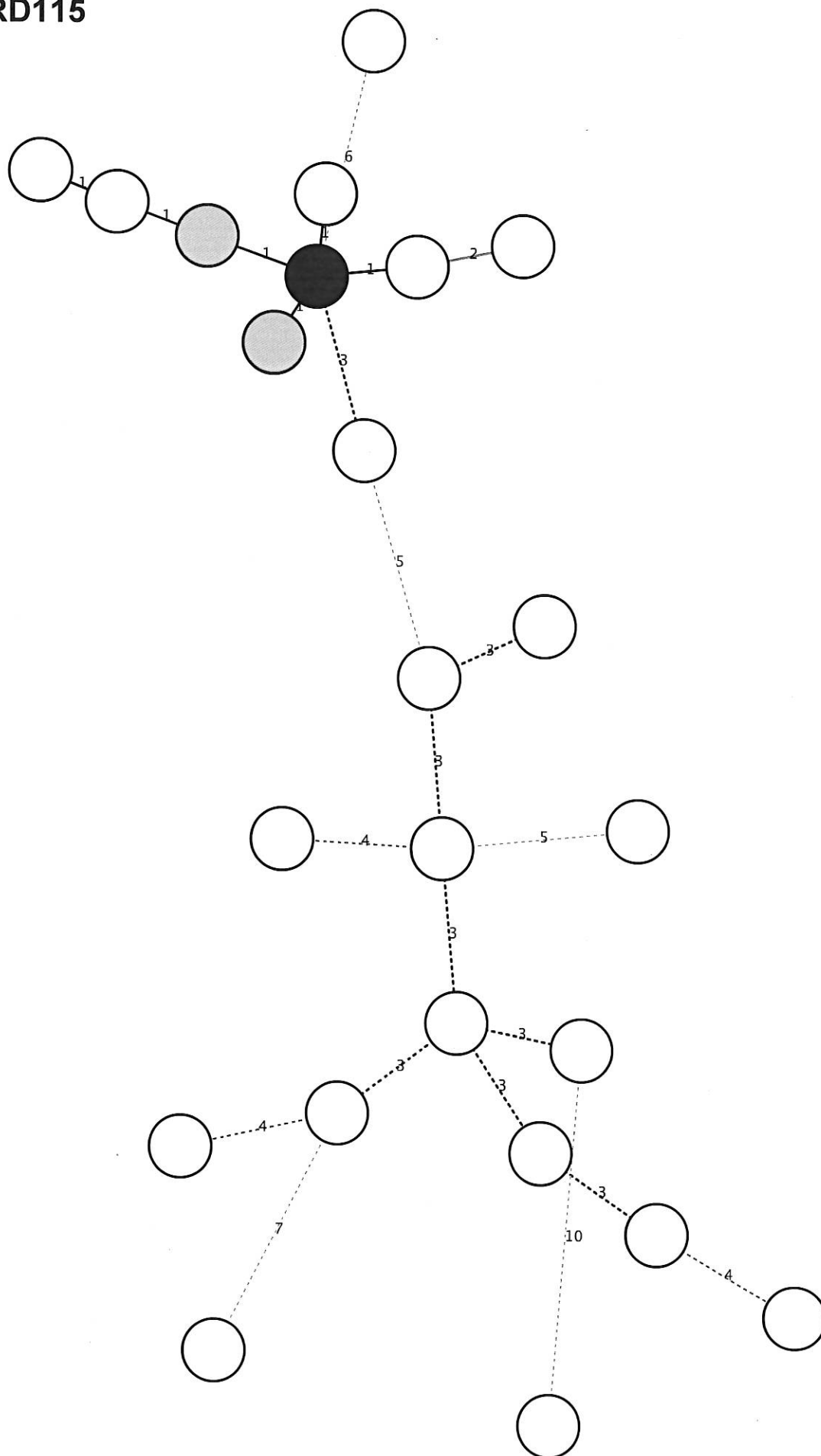

RD726

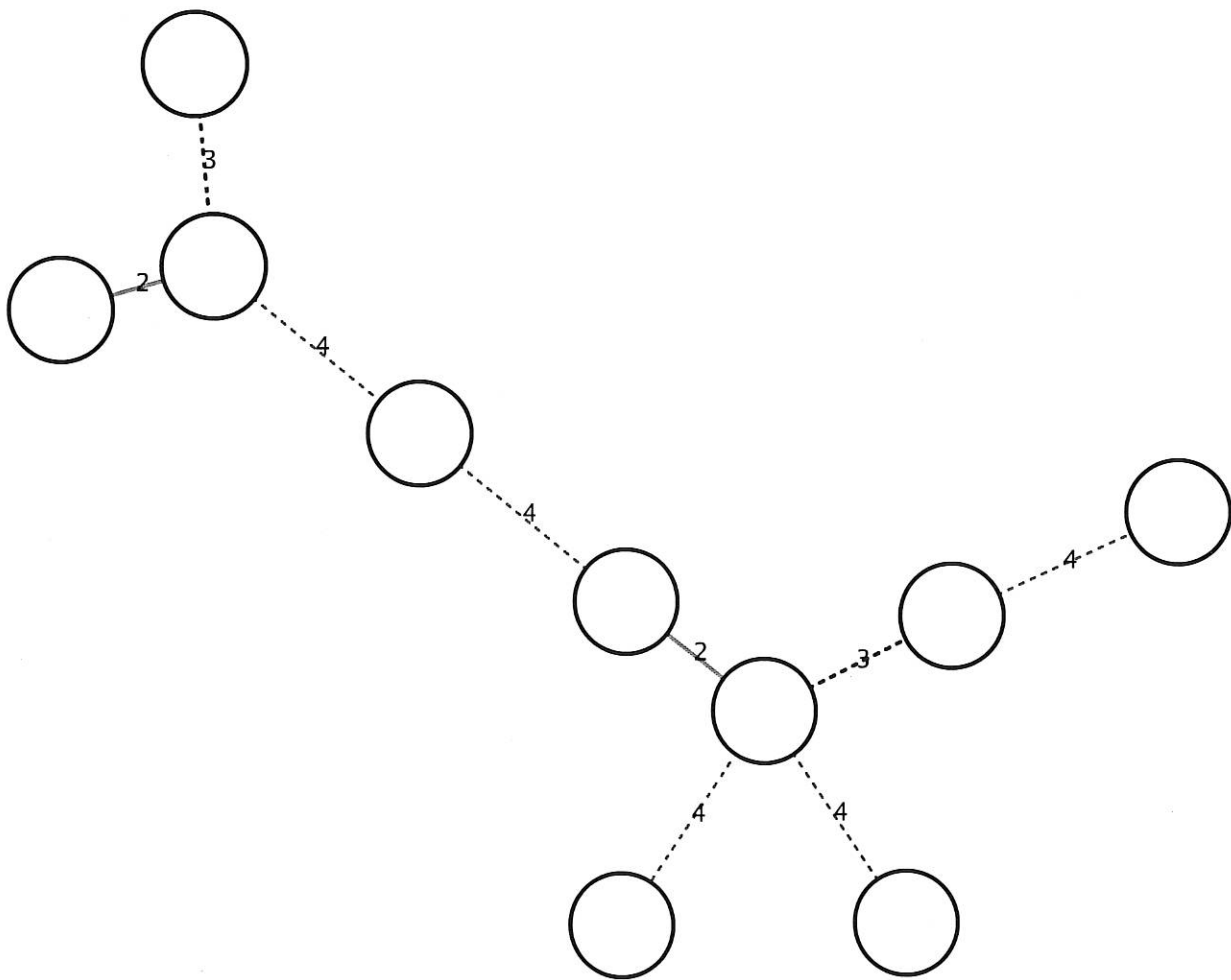

RD219

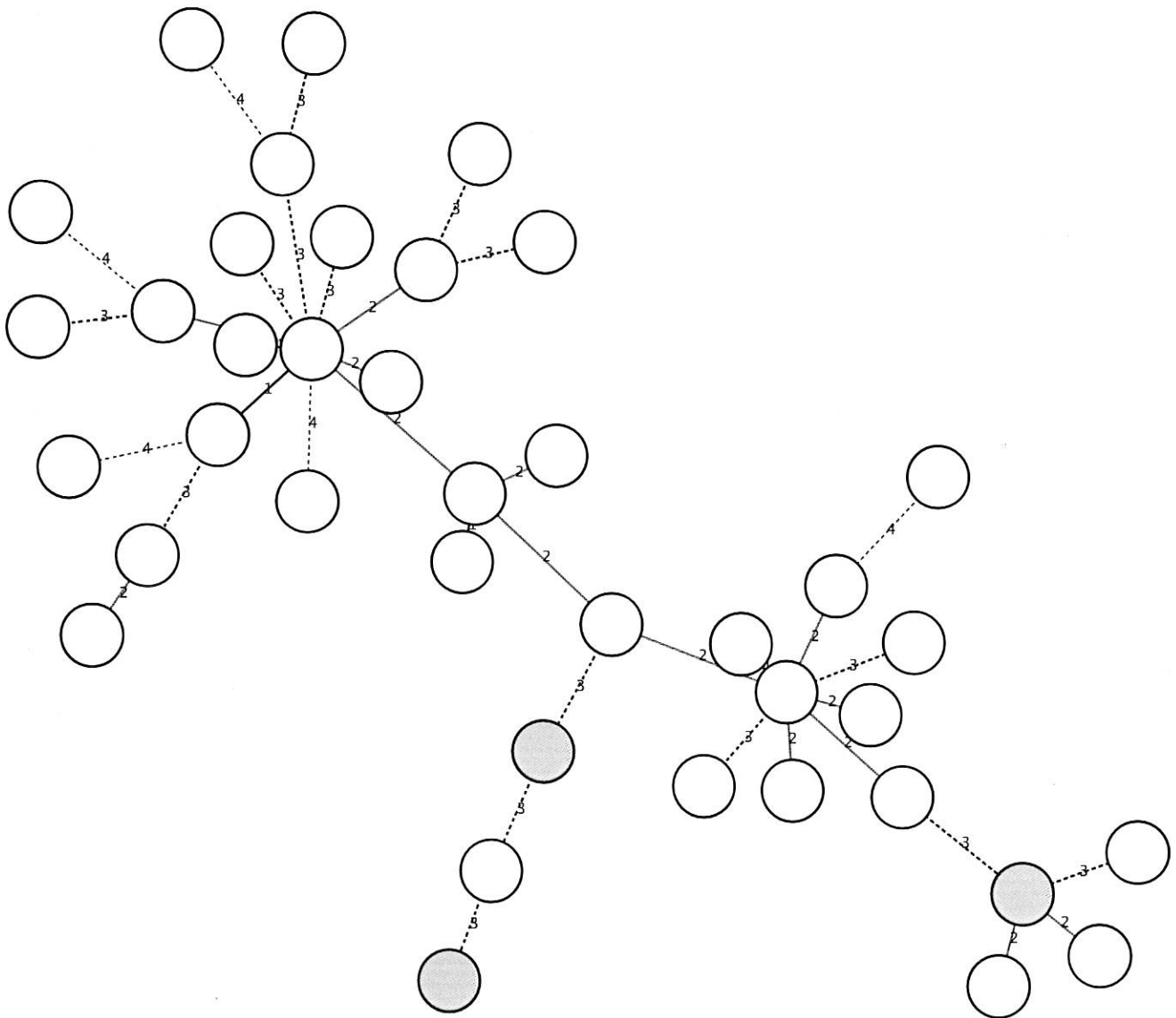

Supplement: Figure S2 — Printouts of the MSTs shown in Figure 2 in which the numbers of allelic differences are shown on the connecting lines. Each empty circle represents a single isolate; dark and light gray circles represent clusters of 3 or 2 isolates, respectively, with identical MIRU-VNTR profiles. (PDF) [file pone.0107150.s002.pdf]

Figure S3

UPCMA-Tree, MRU+HYPR [15] Categorical (L), Spotlog: Categorical (L)

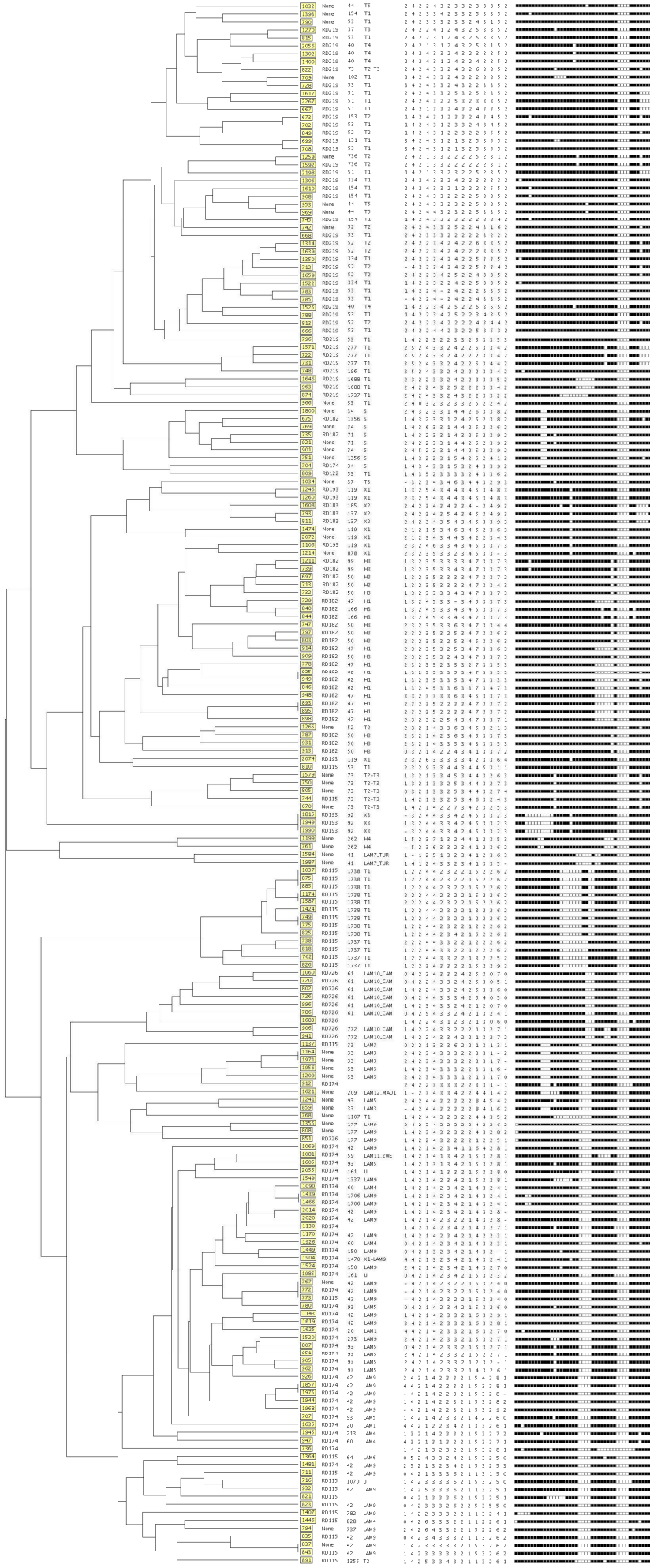

Supplement: Figure S3 — Dendrogram based on combined MIRU-VNTR and spoligotyping data from 197 Euro-American M. tuberculosis isolates bearing deletions RD115, RD122, RD 174, RD182, RD183, RD193, RD219 and RD726 or with no deletion. The dendrogram was generated using the UPGMA method by the MIRU-VNTRplus web application available at www.miru-vntrplus.org. The columns 1 to 6 on the right of the dendrogram represent respectively: 1) isolate ID code (boxed); 2) RD deletion; 3) the SIT (Spoligotype International Type) number; 4) the spoligotype subfamily; 5) the VNTR profiles expressed as a string of 15 numbers, each representing the number of tandem repeats (TR) at a given VNTR position, in the order stated in the paper; 6) the spoligotype binary profile, given as black and white boxes indicating the presence and absence, respectively, of the specific spacer at position 1 to 43 in the DR locus. (PDF) [file pone.0107150.s003.pdf]
